# Twelfth Annual Meeting of the Mississippi Valley Association of Dental Surgeons

**Published:** 1856-04

**Authors:** 


### TWELFTH ANNUAL MEETING OF THE MISSISSIPPI VALLEY ASSOCIATION OF DENTAL SURGEONS.

The Association met, according to adjournment, at 10 o'clock, Wednesday, February 20th, 1856.

Present: Drs. Hamill, Griffith, Goddard, James Taylor, Bonsall, Smith, Ulrey, Taft, Watt, Leslie. Dr. Hamill, Vice President, in the chair.

On motion, Drs. Goddard and Bonsall were appointed to fill vacancies in the examinating committee.

On motion, Dr. Griffith was appointed to fill the vacancy in the executive committee.

The examining committee submitted the following report:

Your committee on membership submit the names of Drs. James O. Martin, Franklin, Ind.; Geo. W. Keely, Oxford, O.; Joseph Richardson, Cincinnati, O.; Edmund Osmond, Port Jefferson, O.; A. G. Stipher, Canton, Ills.; J. B. Newbrough, Dayton, O.; J. H. Bancroft, Mo.; D. W. Harris, West Liberty, O.; E. Bray, Evansville, Ind.; Richard Peckover, Paris, Ky.; J. F. Mercer, Canada West., as candidates for membership.

W. H. Goddard,

J. Taft,

C. Bonsall, } *Committee.*

The report was accepted, and on motion, each name was balloted for separately, when all were declared duly elected by a unanimous vote. On motion,

"*Resolved*, That all members of the profession, medical or dental, who visit our meetings, be invited to participate in the discussions."

A letter from the president, Dr. A. S. Talbert, explaining the cause of his absence, was received and read.

Dr. Leslie, on account of inability to attend all the meetings of the association, tendered his resignation as secretary, which, on motion of Dr. Goddard, was accepted. After balloting to fill the vacancy, and for the ensuing year, Geo. Watt was declared duly elected secretary.

The executive committee presented the following report, which was read and adopted :

Your committee respectfully submit the following report as the order of business :

I. Report of Examining Committee.

II. Report of Officers.

III. Reports of Committees.

IV. Addresses and Essays.

V. Miscellaneous Business, as follows :

1. Does continuous gum-work bear the test of experience ?

2. What is the best method of preparing gold for filling teeth ?

3. What is the best method of drying cavities previous to filling; also, partially filled cavities, when accidentally wet with saliva before the work is completed ?

4. Has block-work any advantages over single teeth, that are not counterbalanced by corresponding disadvantages. If so, what are they ?

5. What are the best methods of, and the best instruments for separating teeth preparatory to filling ?

6. How can the color of the natural teeth that require filling, be most effectually preserved ?

7. Is gutta percha reliable as a base for artificial teeth; if so, the best method of applying it?

8. Can artificial teeth, when mounted on a porcelain base, be worn with advantage in any case?

9. What are the various pathological conditions of inflamed dentine, and what circumstances modify its treatment?

10. What is the modus operandi of arsenic, chloride of zinc, nitrate of silver, creosote, tannin, &c., when applied to inflamed dentine?

H. R. Smith,

J. P. Ulrey.

Sam'l Griffith, } *Committee.*

The examining committee having previously reported, the reports of officers were called for.

Treasurer's Report.

The treasurer would report the following statement of his account, and request that an auditing committee be appointed to examine the same:

| | | |
|---|---|---|
| The balance in the treasury at last account, - | | $194 26 |
| Received since that report, - - - | | 57 00 |
| | | $251 26 |
| By cash paid to G. Watt for essay, - | $100 00 | |
| " " " Brown & Sherwood, ext. inst. | 50 00 | |
| " " " Janitor, - - - | 5 00 | |
| " " " Case, ordered by society, | 12 25 | |
| " " " Joiner's work, painting case, &c., - - - - - | 18 55 | |
| " " " For printing essays, - | 53 44 | |
| " " " For copy-right of essay, - | 1 00 | |
| | $240 44 | |
| Leaving a balance in the treasury of - - | | $11 02 |

All of which is respectfully submitted.

Chas. Bonsall, *Treas.*

Drs. Watt, Goddard and Taylor, were, in accordance with the above request, appointed an auditing committee, and reported as follows:

Your committee, appointed to audit the treasurer's account, respectfully report that they have examined the same, and find it correct.

GEO. WATT,

W. H. GODDARD, } *Committee.*

JAS. TAYLOR,

On motion, adjourned till 2 o'clock, P. M.

AFTERNOON SESSION, 2 O'CLOCK, P. M.—Met according to adjournment. Minutes read and approved.

Present: Drs. Hamill, Goddard, Griffith, Smith, Taft, Bonsall, Osmond, Stipher, Harris, Newbrough, Taylor, Ulrey, Martin, Keely, Bray, How, Richardson, Bancroft, Leslie, Watt, and a number of others not members of the society.

On motion of Dr. Taft, resolved that the name of the "Examining Committee" be changed to the "Committee on membership."

The reports of committees being called for, the committee on microscopic examinations reported as follows:

Your committee on microscopic examinations respectfully report that, some progress is made in the fulfilment of the work assigned to them, but have nothing in proper form to present to the society, but intend to pursue the investigations as opportunity may afford.

J. TAFT,

JAS. TAYLOR, } *Committee.*

A. M. LESLIE,

The report was, on motion, accepted, and the committee continued.

The committee on the prize essay presented the following report:

The committee on the prize essay respectfully report that, according to the instructions of the association, three hundred copies of the essay were printed and distributed to the profession. But one copy has been revised and returned. That one contained important suggestions in regard to language and style. It was received from Dr. Newbrough, of Dayton, O. These suggestions are mostly adopted; and aided by various literary gentlemen, the committee carefully revised the whole essay, and the corrected copy is herewith transmitted. Respectfully submitted.

GEO. WATT,<br>

J. TAFT<br>

JAS. TAYLOR. } *Committee.*

The committee on the society's cabinet reported as follows:

The committee appointed to procure a case for the preservation of teeth, models and other specimens possessed by the society, report that they have procured a case, in all probability large enough for the society's purposes for several years to come. In it they have placed the articles in the hands of the late secretary, A. M. Leslie, viz:

200 specimen teeth, presented by Jones, White & Co.;

1 Under set of teeth, in gutta percha, after Hill's mould, presented by Dr. W. R. Webster.

1 Dog-fish upper jaw, presented by Dr. W. R. Webster.

1 Cast of cleft palate, " " "

2 Casts illustrating absorption, " "

4 " (2 sets) " articulation, " "

1 Full set of teeth, on tin, presented by Dr. J. W. Baxter.

1 Silver stone holder, for polishing plugs, presented by Dr. Peebles.

1 Cast, showing great interalveolar space, presented by Dr. A. M. Leslie,

1 Cast of a mouth with a supernumerary tooth, presented by Dr. A. M. Leslie.

1 Cast, minus the lateral teeth, presented by Dr. A. M. Leslie.

1 Cast, full set, exhibiting the friction of tobacco pipe, presented by Dr. A. M. Leslie.

1 Cast, showing union of central and lateral incisors, with transposition of lateral, presented by Dr. A. M. Leslie.

Your committee suggest, an effort be immediately made to fill up the shelves of the case, by every member preserving and contributing curious casts, teeth and other specimens.

A. M. LESLIE,<br>

C. BONSALL,<br>

JAS. TAYLOR, } *Committee.*

Dr. Bonsall reported, that the faculty make no charge for the use of room for the cabinet of the association.

The committee on dental progress, presented their report, which was received, adopted and ordered to be published with the minutes.

REPORT ON DENTAL PROGRESS.

*To the Mississippi Valley Association of Dental Surgeons.*

GENTLEMEN,—The undersigned members of your Committee on Dental Progress, respectfully report that though aware of the difficulties to be encountered, and the responsibilities involved in the performance of the duties assigned, and conscious of their inability properly to discharge these duties, yet they have endeavored, as far as talent and circumstances would admit, to fulfill the requisitions imposed on them.

By a resolution of the association, at its last meeting, the committee was instructed to embrace, in the report, the progress of the three years preceding the present date; but, from circumstances not necessary to mention, this became impracticable, and we accordingly confine ourselves to the past year, as originally intended by the society.

The association never having received and adopted a report, we have no means of ascertaining the traits of character desired by it. Left, therefore, to our own resources and suggestions, we offer you the following, relying on your kindness to excuse its imperfections, and your judgment to correct its errors.

The progress of dental, like that of general science, is necessarily irregular. A first principle becomes implanted in the mind of an individual member of the profession, it germinates, springs up, and, in turn, yields its fruits in the development of scientific truths adapted to our professional wants.

As in the great kingdom of nature, the earth yields more abundantly in some seasons than others, as it brings forth, at the same time, sweet fruits and bitter, as it produces, from the same locality, the healthful medicine and the virulent poison; so our professional soil is prolific or barren, so it yields important truths or baneful errors, according to the character of the cultivation, the quality of the seed sown, or the nature of the mental soil in which it is cast.

In estimating the crop, the chaff and the tares must be separated from the grain; and so in estimating the progress of our profession, it is necessary to analyze the newly advanced ideas, that the worthless, and especially the hurtful, may be distinguished from the useful. In a word, the retrogression must be deducted from the progression, to ascertain the real advancement made.

Since our last annual meeting, the "American Society of Dental Surgeons," the parent association, held its annual meeting in this hall, the minutes of which you have doubtless all read. Simultaneous with said meeting, this association held a called meeting, the prominent object of which was to welcome the parent society to the West. The harmony and good feeling manifested by the two societies, and by members of the profession individually, cannot fail to produce a happy and lasting effect on the welfare and prosperity of the profession. All present, we believe, will join in the sentiment, "It was good to be here." The society had under consideration the question of its final dissolution, which was still pending when it adjourned. At a subsequent meeting in Philadelphia, the question was again postponed. The only arguments used, we believe, in favor of dissolution were, that the progress of the profession had rendered such an organization no longer necessary; that it had served the purpose for which it was organized; and, that a new organization, on a more liberal basis, would better subserve the present wants of the profession.

State and local societies have, we believe, in general, manifested more than ordinary vitality, especially the "Pennsylvania Association," if we may judge from the number of its meetings, and the reports of its discussions. That these discussions have not elicited, in all cases, correct conclusions, is, perhaps, only what is to be expected while man is mortal.

But the most prominent event, in the way of association, is the "American Dental Convention," which met in Philadelphia August 2d, 1855, at the call of some sixty of the dentists of Philadelphia and vicinity. Articles of association were adopted, and signed, by some eighty members of the profession, representing the following states and territory, viz.: Lousiana, Delaware, Kentucky, Ohio, New Jersey, Connecticut, Massachusetts, New York, Pennsylvania, and D. C.

The discussions, as reported, embrace a considerable range of subjects, and a very free interchange of sentiment was elicited. (See October Nos. of *News Letter* and *Dental Register.*) We regard this step as a decided advance in the way of association, and hope that the next meeting will be far more numerously attended; in short, that it will receive the attention from the profession which it merits.

The *American Journal of Dental Science*, the pioneer in this field, holds its own, pursuing "the even tenor of its way." As far as we are aware, it has undergone no special change during the past year. As usual, it keeps pace with the progress of the profession, and is still the largest Dental Journal extant.

*The Dental Register of the West* has donned a new suit, and is greatly enlarged, each number now containing one hundred and twelve pages. From the contents of the two enlarged numbers already issued, we infer that there will be no lack of material to fill it.

The *Dental News Letter* is regularly issued, gradually and steadily improving in appearance, and in the character of its contents. It happily adapts its size to its circumstances, enlarging when pressed by important matter. We hope it will never have occasion to *grow less.*

The *Dental Recorder* is still promptly published, and manifests a decided spirit of progress. Being issued monthly, it is often able to furnish us with new and important ideas, in advance of all its co-laborers.

The *Dental Obturator*, though started previous to our last meeting, has principally made its acquaintance with the profession since. It gives evidence of the vitality and energy which we have a right to expect from its nativity and parentage. We hail it as a valuable acquisition to our periodical literature.

A year ago, a chair of "Chemistry and Metallurgy" was established in the "Ohio College of Dental Surgery," which, it is hoped, renders the course of instruction more thorough and satisfactory to all concerned. Additions have been made to the cabinet in the shape of morbid specimens, enlarged models, anatomical preparations, etc., all, it is hoped, tending to facilitate the cause of dental education.

We regret that we are unable to give you a definite account of the past annual progress of the other colleges. The chairman of the committee addressed, by mail, the Deans of the respective faculties, soliciting information on this subject. No answer is yet received. (A note has been received from Prof. Arthur, of the Philadelphia College, since the meeting of the association, reporting, briefly, an increased class, facilities for teaching, etc., and expressing his regret that he had not leisure to report definitely.)

In the literature of the profession, aside from the periodicals, there is not much change. A new edition of "Harris's Principles and Practice of Dental Surgery," and a new and improved edition of "Fox and Harris," constitute about all that is new in this line. Both these works are published by Lindsay and Blakiston, in their usual fine style.

In the general state of professional intercourse, there is great improvement. This progress was enhanced, no doubt, by the joint meeting of this and the "American Society," and by the "American Dental Convention." When members of the profession meet personally, freely exchange ideas, and socially entertain each other, they become better pleased with themselves and with one another, and, as a natural result, jealousies vanish, and misunderstandings are corrected.

The improvement in this respect is evidenced by even a casual glance at the journals. The prolonged and disgusting quarrels, the personal abuse and vituperations, are now seldom seen in their pages. We are glad to be able to state that articles of the class referred to, if written, have been, except in a few cases, for the past year excluded from the periodicals.

In the preceding remarks, we have endeavored to sketch the general progress of the profession, and, conscious of our inabilities in this, we advance with diffidence to the consideration of special improvements.

As far as we are able to judge, there has been a gradual improvement in the "continuous-gum work" since our last meeting. The alloy of platinum and arsenic for soldering platinum is worthy of attention.

Of the progress made in Dr. Loomis' style of work, we are not prepared to speak. We have seen some specimens out of the mouth, which are very handsome, but we have had no opportunity to examine practical work mounted by this method.

Something similar in style is the work of Dr. Sattersthwaite. The plate and gum are moulded and carved from suitable porcelain paste, single teeth are inserted and the whole baked at the proper temperature. This also looks well out of the mouth, we have had no opportunity of observing it in practical use. From the college cabinet we are able to present for your inspection, a specimen of each, We fear that neither will stand the test of time and experience, but we wish the gentlemen respectively the most abundant success. Both we believe are secured by letters patent.

A new or modified, style of work, mounted on a base of gutta percha, has for a few months, reeeived, and is still receiving considerable attention from the profession, We have made no practical test of the work. It is modestly offered for temporary work only, with an intimation that it will one day be relied on for permanent operations. In all previous dental experiments with gutta percha, the color has been an objection. In this the gum color is more life-like, and is said to be permanent in the mouth. The inventor, Dr. Slayton, has secured himself on his method of refining and coloring the gutta percha. We have not ascertained what coloring agent or agents, he has used. A lifelike and permanent gum color may be imparted to pure gutta perchia by a solution of carmine in chloroform. Dr. S. has spoken for himself through the journals to which we refer you for particulars.

But one article of crystal gold is at present in market. We refer to that of A. J. Watts & Co. It is vastly improved in color, structure, and tenacity, and in all respects calculated to increase its practical utility. Its improved color is due to its crystaline structure, and not to any increased purity, for it was pure gold before. The dark colored specimens formerly in market were admirable preparations of *sponge* gold but, in the main, *they were not crystaline.* Gold crystals, whether large or small, if perfect, will give the same color, for the face of a crystal is always a plane surface, and its boundaries are rightlines. That similar surfaces will have the same influence on light will be admitted by all who think of the subject. We regard this article in its present improved state, as a great acquisition, and an admirable article for filling teeth, notwithstanding the recent unanimous report of a large committee against it.

The drying of cavities preparatory to filling elicited considerable discussion at some of the professional meetings of the past year. We know of nothing new on this head, unless it be the application of the warm air bath, as suggested in the October number of the "Dental Register."

The production of anasthæsia by the compression of the carotids has elicited some attention. We cannot speak ex-[illegible]m entally of its practical applacation to dental surgery but those who have tested it, are favorably impressed. For particulars and manipulations, see "Dental Record[illegible]," Vol. IX. page 249 and other journals.

The application of cold to produce local insensibility is also receiving attention, especially in Great Britain. (See a discussion of this subject in the "Dental Register" for January, 1856, and other journals). We hope that this, and the compression of the carotids will receive close attention.

In the manufacturc of dental instruments we are aware of no very striking change. The spirit of progress still prevades this department, and through the talents and energy of Messrs. Sherwood, Chevalier, Kern and others it is not likely to fall behind.

In the manufacture of teeth the usual progress is manifested. Indeed this department seems to advance by geometrical rather than arithmetircal progression. Orders are filled in accordance with the judgment of the most thorough and the fancy of the most fastidious. The well known firm of Jones, White & McCurdy, are still in advance of all competitors, and as usual, furnish us with almost everything that could be desired in the shape of artificial teeth. Messrs. Orum & Armstrong also furnish most beautiful teeth, of almost every desirable shape and color. Their systematical methods of numbering their moulds and the shades of their teeth is a convenient arrangement, enabling the practitioner to select by order as satisfactorily as by personal attention.

For want of information we have doubtless failed to notice many improvements. These we hope, will not be overlooked by the society.

In all the departments thus far noticed the progress is encouraging. But there is a drawback. Ever since the *wise* men came from the *east* to Jerusalem," we are prone to regard the east as the place whence wisdom springs. As the orb of day arises to refresh and beautify the earth, it is little wonder that the poor pagan bows down at his coming, and "worships toward the east." Nor is it any wonder that his devotions become less fervent and his faith less firm, when his prayer is answered by the blasting and mildew of the east wind.

So the light of our profession the star of its progress arose in the east, and the profession in the west have worshipped toward it and made pilgrimages to its temple. Its course still upward, it illumed the west gladdening the hearts of its devotees. But a breeze is felt, poisonous as that which blasted the corn of Pharaoh's dream, and yet "*wise* men from the east" advise us to breathe it.

But to drop the metaphor, we report with regret, that *some* of the prominent members of the profession east are advocating amalgam fillings. The standing, age and influence of some of these enable them to exert a very deleterious influence on the cause of dental science, an influence whose effects they will scarcely be able to counteract when their eyes again become opened. The occasion of this retrogression is the introduction of a supposed new amalgam formula, which it is thought, cannot tarnish in the mouth, the oxydation being prevented merely by the addition of another base metal to the compound. When we reflect on the past course and present position of these men we can only say "How are the mighty fallen!" Believing that their own good sense will prove sufficient to reclaim them, we regard even this as but a temporay retrogression.

In conclusion, we state as an act of justice, that the member of your committee whose name is not signed to this report is responsible for nothing it contains. His publication, *on his own responsibility*, of a document which we understood to be referred to the committee, without discussion and when but partially read, placed it beyond our power to act in concert with him. Respectfully submitted.

GEO. WATT.
H. R. SMITH.

The reading of essays being next in order, a letter was received from Dr. How, stating reasons for not being able to fulfil his appointment, which were accepted, and his appointment continued.

Dr. Smith read an essay on "Dental Education," which, on motion of Dr. Goddard, was requested for publication. Dr. Ulrey was excused and continued.

On motion, the election of officers was made the order of the day, for 2½ P. M., to-morrow.

Moved, by Dr. Taft, that — copies of the essay be published by the Society.

On motion, a committee of three were appointed to obtain subscriptions for said essay.

Drs. Taft, Bonsall, and Bray, said committee.

On motion, the price of the essay was fixed at six cents in paper, — in cloth.

Drs. Taft, Taylor, and Ulrey were appointed a committee to superintend the stereotyping, printing, and publication of the essay.

Proceeded to the discussion of Item No. 1, of miscellaneous business, which was participated in by Drs. Taylor, Ulrey, Keely, Taft, Horton, Goddard, Griffith, and others.

Item No. 2 was taken up and discussed by Drs. Goddard, Bonsall, Smith, Ulrey, Keely, Richardson. Taft, Leslie, Horton, James, and others.

On motion, adjourned to meet at 8 o'clock, A. M., to-morrow.

THURSDAY, Feb. 21st, 8, A. M.

Met according to adjournment. Present, Drs. Bonsall, Smith, Ulrey, Bancroft, Stipher, Osmond, Taft, Hamill, Grffith, Goddard, Bray, Richardson, Taylor, Keely, Watt, and others, not members of the Society.

Minutes read and adopted.

The committee on subscriptions to Prize Essay reported that three thousand copies are subscribed for. Report accepted and committee continued.

Proceeded to miscellaneous business. No. 3 was discussed by Drs. Taylor, Bonsall, Smith, Ulrey, Watt, Taft, Griffith, Goddard, and Horton.

No. 4 was taken up and discussed by Drs. Goddard and Bonsall.

No. 5 was taken up and discussed by Drs. Griffith, Bonsall, Goddard, Bray, Ulrey, Keely, Hamill, Horton, Ramsay, Leslie, Taft, and Taylor.

On motion, adjourned to meet at 2, P. M.

AFTERNOON SESSION, 2, P. M.

Met according to adjournment. Present, Drs. Bonsall, Griffith, Goddard, Bray, Taft, Ulrey, Bancroft, Smith, Osmond, Stipher, Newbrough, Hamill, and others of the profession not members.

Minutes read and adopted.

No. 6 of miscellaneous business was discussed by Drs. Bonsall, Griffith, Goddard, and Taft.

The election of officers being the order of the day, Drs. Taft and Ulrey were appointed tellers.

After balloting, the following was found to be the result:

*President*—Dr. J. B. Newbrough; *Vice President*—Dr. E. Bray; *Cor. Sec'y*—Dr. James Taylor; *Treasurer*—Dr. Charles Bonsall.

*Executive Committee*—Drs. J. Taft, J. P. Ulrey, J. Richardson.

*Committee on Membership*—Drs. James Taylor, H. R. Smith, J. Taft.

*Committee on Dental Progress*—Drs. Geo. Watt, H. R. Smith, J. Taft.

Drs. Osmond, Keely, and Bray were appointed to read essays at the next meeting, in addition to those previously appointed.

The Executive Committee requested members and others to suggest, at any time previous to the next meeting, subjects which they desire to hear discussed, under the head of "miscellaneous business."

The Committee on Dental Progress also requested all members of the profession to forward improvements and items of progress, without waiting for further notice.

Vice President Hamill vacated the chair in favor of the

President elect, who, on account of imperative engagements elsewhere, immediately called the Vice President elect to take his place.

No. 7 of miscellaneous business was taken up and discussed by Drs. Taylor, Goddard, Bonsall, Ulrey, and Taft.

No. 8 was discussed by Drs. Taylor and Horton.

No. 9 was taken up, when, on motion, Dr. Hamill read an essay on inflammation of dentine, as an introduction to its discussion, which was, on motion, requested for publication.

On motion, adjourned to 6½ this evening.

EVENING SESSION, 6½ P. M.

Met according to adjournment, Vice President in the chair, Present, Drs. Bonsall, Taylor, Hamill, Osmond, Richardson, Taft, Bancroft, Bray, Smith, Ulrey, Griffith, Stipher, Harris, Watt, and others.

Minutes read and adopted.

The discussion of No. 9 was resumed and participated in by Drs. James Taylor, Griffith, Richardson, Taft, Horton, Ulrey, Joseph Taylor and others.

No. 10 was discussed by Drs. Bonsall, Ulrey, Watt, Richardson, Taft, Taylor, and Griffith.

By request, Dr. Goddard reported an interesting case of tumor, or preternatural growth of the gums, a full report of which was requested for publication in the Dental Register.

On motion of Dr. Taft,

*Resolved*, That we appropriate $15 to $20, for the procurement of wood cuts to illustrate the same.

On motion,

*Resolved*, That the Treasurer be authorized to pay $5 to $10 for a synopsis of this day's discussions, for publication in the Register.

On motion of Dr. Goddard, the Treasurer was ordered to pay the janitor $5 for his services.

On motion, the Treasurer was directed to notify delinquent members in regard to the state of their accounts with the Society.

The thanks of the Society were returned to Dr. Hamill, for the able and impartial manner in which he presided, and to the College Faculty for the use of the Hall, &c.

On motion of Dr. Bonsall, adjourned to meet February 25th, 1857, at 10 o'clock, A. M.

